# A self-made suspension trainer as a tool for core muscle activation and motivation in a girl with cognitive and motor impairments: a case report

**DOI:** 10.3389/fresc.2026.1658283

**Published:** 2026-02-25

**Authors:** Volodymyr Maksym

**Affiliations:** Independent Researcher, Lviv, Ukraine

**Keywords:** case report, core stability, low-resource intervention, motivation, neurodevelopmental disability, pediatric rehabilitation, suspension trainer

## Abstract

**Background:**

Children with combined cognitive and motor impairments often experience persistent difficulties with postural control, core muscle function, and motivation for rehabilitation. Low-cost, engaging approaches may be particularly relevant in resource-limited settings.

**Case presentation:**

This case report describes a 13-year-old girl with a history of perinatal hypoxic-ischemic brain injury and early-onset seizures, who had severe cognitive and motor impairment. Caregiver-provided clinical documentation indicated severe intellectual disability (ICD-10: F72.0) and degree II–III activity limitations (as graded within national rehabilitation documentation) across domains including self-care, mobility, communication, behavioral regulation, and learning-related functioning. Clinically, she demonstrated marked core and scapular weakness, a rounded upper back posture, and reduced motivation after years of conventional therapy.

**Intervention:**

A self-made suspension trainer was constructed from accessible materials (rope, gym stick, and floor mat) and integrated into therapy sessions. The main task consisted of supported sit-ups combined with assisted pulling, enabling partial weight support, with gradual progression in repetitions and independence.

**Outcomes:**

Across sessions, observable changes were noted in task performance and engagement, including increased repetitions and reduced need for assistance. Outcomes were documented through session logs, therapist observation, and caregiver report; no standardized outcome measures were applied.

**Conclusion:**

This single-case report provides qualitative, hypothesis-generating observations suggesting that a low-cost, therapist-made suspension trainer may support engagement and task performance in a child with complex neurodevelopmental needs. These findings are not generalizable and warrant evaluation using standardized outcomes and longer follow-up.

## Introduction

Children with combined cognitive and motor disabilities often present with impaired postural control, proximal muscle weakness, and difficulties in motor planning, which significantly limit their participation in daily and therapeutic activities (1). Conventional pediatric rehabilitation typically relies on repetitive, therapist-led routines that, while functional, may reduce child engagement and intrinsic motivation over time (2). Motivation is increasingly recognized as a critical factor in rehabilitation outcomes; interventions that introduce variety, sensory stimulation, and meaningful tasks tend to sustain engagement and support functional gains in children (3).

In settings with limited resources, low-cost or improvised therapeutic tools offer vital clinical utility when specialized equipment is inaccessible (4). Moreover, core stability or suspension-based interventions—by providing unstable support surfaces—can promote proprioceptive and vestibular inputs, potentially enhancing postural control and core muscle activation (5, 6). Such interventions combine physical challenge with novelty, which may boost motivation.

This case report describes the application of a therapist-constructed suspension trainer, made from accessible materials, to enhance core muscle activation and motivation in a 13-year-old girl with complex neurodevelopmental needs. To our knowledge, no prior reports have documented the use of a simple suspension device in pediatric rehabilitation within a resource-constrained context.

## Case presentation

The patient was a 13-year-old girl with a history of perinatal hypoxic–ischemic injury and early-onset seizures, presenting with severe cognitive delay and motor impairment affecting ambulation, balance, and coordinated movement.

On clinical examination, she demonstrated marked weakness of the core and scapular musculature. A stooped posture with rounded shoulders and exaggerated thoracic kyphosis was evident, largely attributable to insufficient upper back muscle activation combined with pectoral muscle tightness. The onset of adolescence and a period of rapid growth further exacerbated postural deviations and increased the complexity of rehabilitation needs.

The patient also displayed reduced motivation toward therapy. Having undergone years of conventional physiotherapy, she exhibited limited engagement, primarily due to the monotony and repetitiveness of traditional exercise routines.

### Baseline clinical and functional profile (from available records)

According to caregiver-provided clinical documentation from mid-2023, the child had a documented diagnosis of severe intellectual disability (ICD-10: F72.0) with major limitations in adaptive functioning and dependence on continuous adult supervision for daily activities. Communication was described as severely restricted, with limited functional speech and reliance on non-verbal means, and attention and self-regulation were reported as unstable. An Individual Rehabilitation Program recorded degree II–III activity limitations (as graded within the national rehabilitation documentation) across domains including self-care, mobility, orientation, communication, behavioral regulation, and learning/education-related functioning. These domains broadly correspond to ICF Activity and Participation areas; however, no formal ICF coding or standardized international outcome measures were applied in the present report. The above information is summarized here in anonymized form.

**Figure 1:**
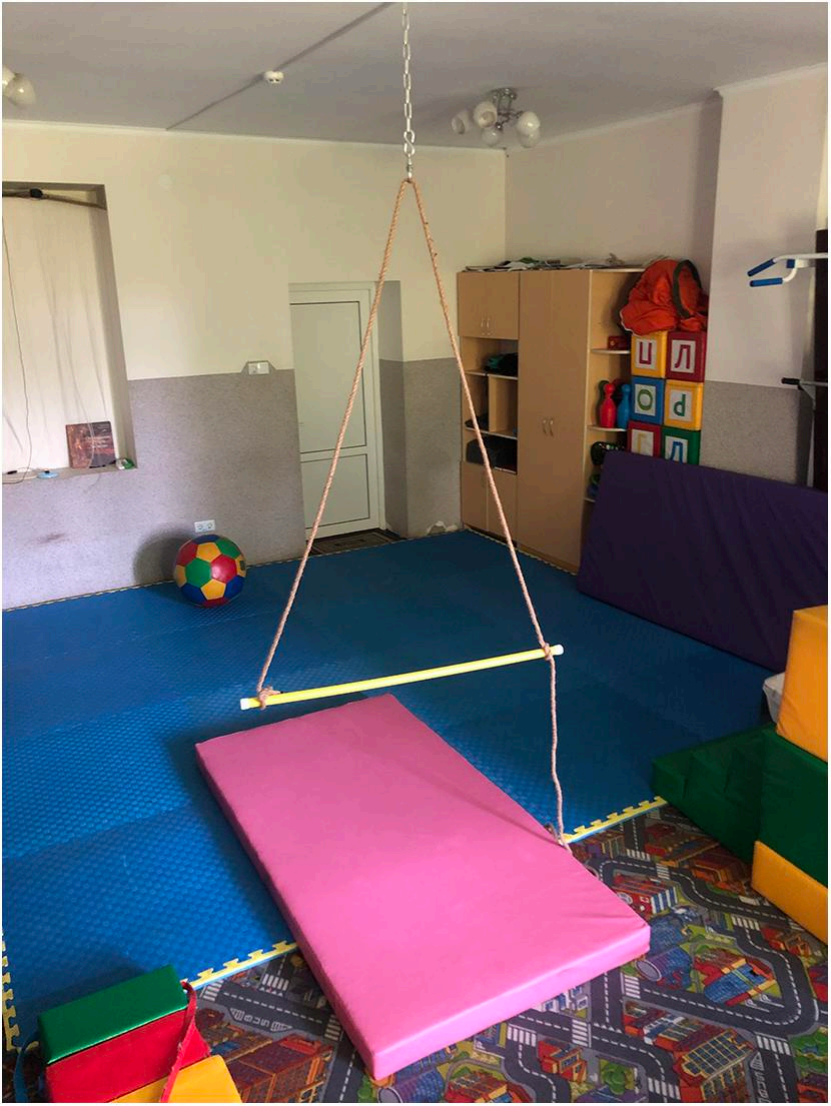
Photograph of the self-made suspension trainer constructed from a rope, ceiling-mounted loop, and gym stick.

**Figure 2:**
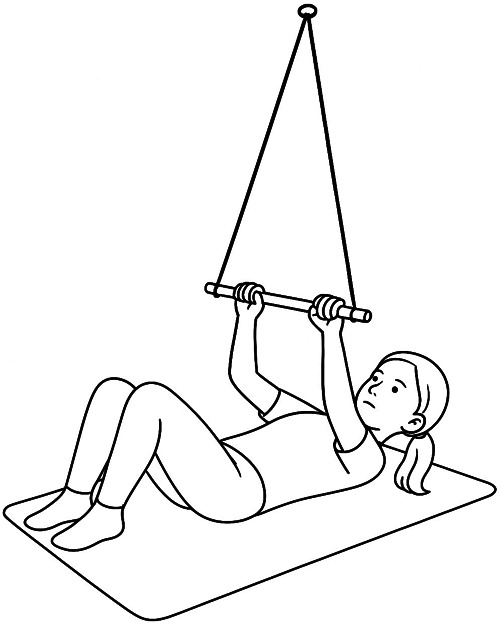
Schematic illustration of the starting position for assisted sit-ups using the suspension trainer (supine position, knees flexed, hands holding the suspended stick).

To address both biomechanical and motivational barriers, a self-made suspension trainer was designed from readily available materials. The device consisted of a rope threaded through a ceiling-mounted loop, with its two ends fixed to the extremities of a gym stick (Figure 1). This configuration allowed the creation of a horizontal support bar that the child could grasp while lying supine (Figure 2).

**Figure 3:**
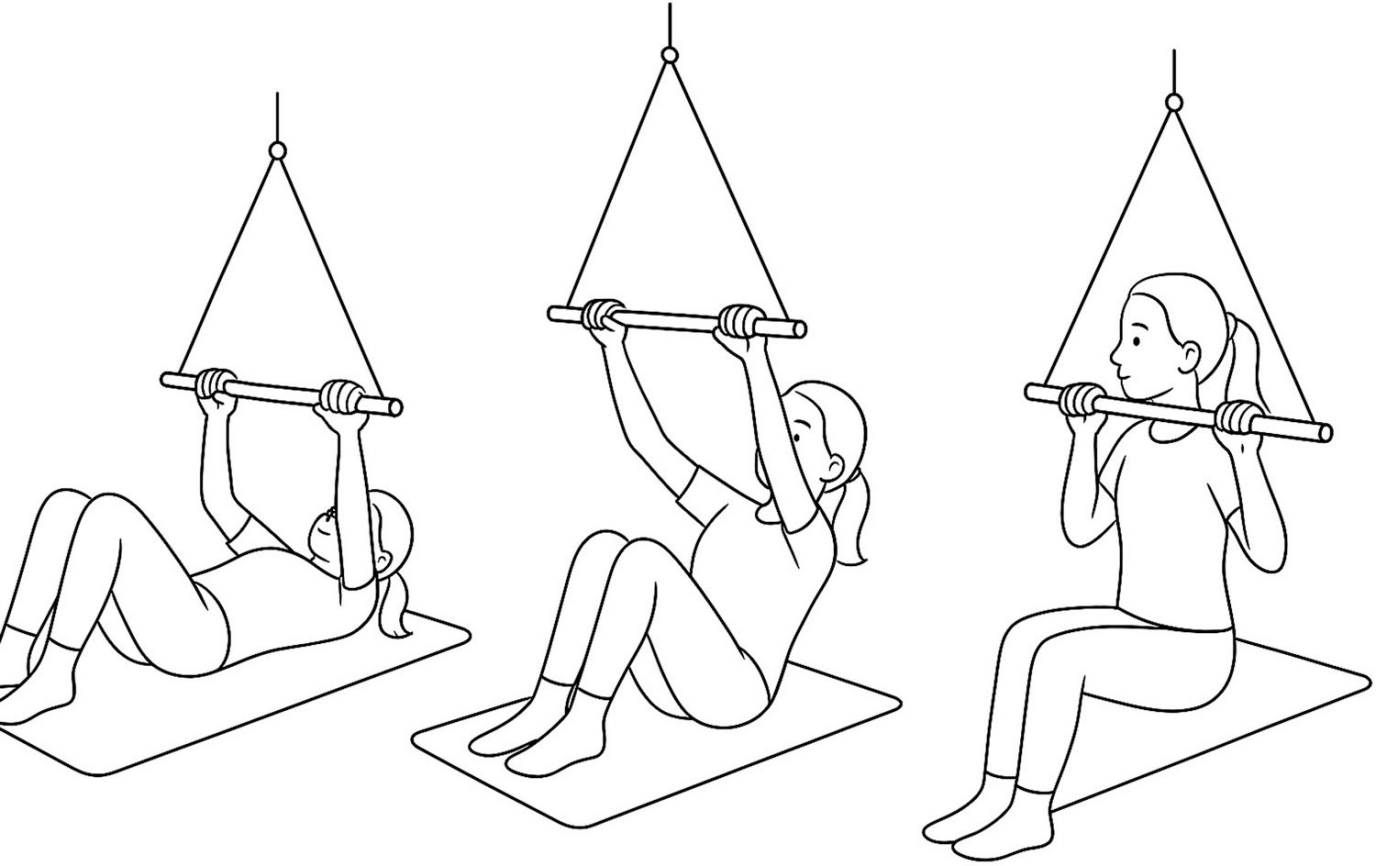
Schematic sequence of the assisted sit-up movement using the suspension trainer: **(A)** supine start position, **(B)** mid-lift phase, **(C)** upright supported sitting.

The exercise involved an assisted sit-up movement in which the child pulled on the suspended stick while activating abdominal and back muscles. The sequence of the movement is illustrated in three phases, from the starting position to the upright supported posture (Figure 3). Initially, the child was able to complete only 1–2 repetitions before fatigue. Over the course of several sessions, her endurance improved, and she successfully performed 10–15 repetitions with minimal assistance. Outcomes were documented through session logs, therapist observation, and caregiver report; no standardized outcome measures were applied.

## Intervention

### Name of intervention

A self-made suspension trainer, informally referred to as the “*Karolina” trainer*, was developed and applied as the central therapeutic tool.

### Rationale

The intervention was designed to provide a more engaging, motivating, and functionally meaningful alternative to conventional core-strengthening exercises. The suspension design allowed the child to integrate larger muscle groups, benefit from assistance-as-needed, and experience dynamic proprioceptive and vestibular stimulation.

Materials and equipment

One durable rope, doubled and anchored to a ceiling hook (previously used for a sensory hammock).
A lightweight gymnastics stick attached horizontally at the rope's ends, forming a suspended bar.
A padded floor mat to ensure comfort and safety.

A schematic line diagram of the setup is provided (Figure 1).

### Provider

The intervention was planned and supervised by a licensed physical therapist.

### Procedures

The participant lay supine on the mat with knees flexed, grasping the suspended bar above the chest.
The exercise consisted of assisted sit-up movements, during which core activation was facilitated by the arms and upper back.
Bar height and grip width were adjusted individually to ensure both safety and progression.
Natural swinging of the rope introduced controlled instability, engaging proprioceptive and vestibular systems.

### Therapeutic principles applied

**Assistance-as-needed:** stronger upper body muscles compensated when trunk muscles fatigued.
**Dynamic instability:** swinging motions challenged balance and sensorimotor control.
**Progressive overload and adjustability:** exercise intensity was gradually increased by modifying repetitions, reducing compensatory support, and later transitioning to classical sit-ups and back extensions.

### Schedule and intensity

Frequency: 2–3 sessions per week.
Duration: each session included 2–3 sets of suspension sit-ups, starting from 1 to 2 repetitions per set and progressing to 10–15.
Total intervention period: 8 weeks.

### Tailoring

Bar height, hand position, and therapist assistance were adapted according to the child's motor ability and weekly progress.
Exercises were progressed from assisted sit-ups to independent classical sit-ups and back extensions.

### Adherence and fidelity

The child's motivation was maintained through playful elements, positive feedback, and progressive goal-setting.
Session logs were recorded by the therapist to document progression and adaptation.

### Modifications and progression

Initially, the participant required considerable arm and back support.
Over the intervention period, compensatory use of the arms decreased, and trunk control improved.
By the end of the program, the participant was able to perform independent sit-ups and back extensions, with changes consistent with improved task performance and trunk control during practice.

## Outcomes

Outcomes were documented through therapist observation, session notes, and caregiver report; no standardized or objective outcome measures were applied. Over the course of sessions, the child progressed from completing 1–2 repetitions with early fatigue to 10–15 repetitions with minimal assistance. Clinically, the therapist observed more stable trunk control during the task, less forward trunk inclination during walking/running attempts, and improved coordination during functional activities. The caregiver also reported increased willingness to participate and reduced avoidance of therapy activities.

Observed changes are summarized in Table 1.

**Table 1:** Functional and motivational changes observed in the patient before and after intervention.

Parameter	Baseline (before intervention)	After intervention
Coordination	Poor; frequent involuntary movements, especially during gait; forward trunk inclination when attempting to accelerate	Therapist and caregiver reported fewer involuntary movements and improved dynamic coordination; reduced forward trunk inclination during walking/running attempts.
Gait and running	Slow and unstable walking; attempts to run accompanied by poor posture and excessive trunk lean.	Able to attempt short bouts of running with improved posture and speed compared with baseline; described by caregiver as “running very well”
Posture	Stooped posture with pronounced rounding of shoulders; habitual slouching.	Less slouching observed; shoulders appeared more retracted; improved upright alignment noted during rest and movement.
Motivation/engagement	Low; often reluctant and avoidant of exercises; preferred play activities.	Higher engagement; more frequently requests sessions and participates more willingly; playful participation increased.

All entries reflect qualitative observations and caregiver report; no standardized measures were applied. In addition to functional gains, caregiver feedback highlighted the psychosocial value of the intervention.

According to the patient's mother:

“I want to thank the physical therapist for identifying the difficulties with coordination, walking, posture, and motivation of my daughter. Thanks to his well-organized sessions and support, her coordination improved, and she not only runs — she runs very well! We are both very grateful to him.”

Such testimony provides contextual information on perceived changes; however, it remains subjective and does not replace standardized outcome assessment.

## Discussion

This case report presents practice-based observations following the use of a therapist-designed, low-cost suspension trainer in a child with neurodevelopmental impairment. Core dysfunction is widely recognized in children with cerebral palsy and related conditions, often contributing to impaired balance, reduced trunk control, and secondary musculoskeletal deformities (1–3). Previous randomized controlled trials confirm that core stability training improves gait, upper limb function, and gross motor outcomes in pediatric populations (1–6). However, many of these programs rely on structured equipment or standardized exercises that may lack adaptability and engagement.

In the present case, the suspension-based intervention integrated therapeutic principles with fitness-derived mechanisms such as progressive overload and dynamic instability. The rope-and-stick construction required active stabilization in multiple planes, offering proprioceptive input consistent with prior evidence highlighting the importance of unstable surfaces and multisensory feedback for postural control (5–7). Importantly, the novelty and play-like nature of the device appeared to reignite the child's motivation, aligning with the broader understanding that emotional engagement and task variability are crucial for sustained rehabilitation adherence (2, 7).

The observed changes in posture and mobility-related task performance, including less slouching and improved walking/running attempts, are broadly consistent with prior reports on trunk/core-focused interventions in pediatric populations (1, 6). Improvements in task performance have also been described in core stability programs targeting upper-limb function in children with cerebral palsy (3). Yet, unlike structured protocols, this approach was embedded in a personalized, context-specific solution requiring minimal cost and no specialized equipment. This may hold particular relevance in resource-limited settings, where access to rehabilitation services and conventional devices can be constrained (4).

### Limitations

This report has several important limitations inherent to its design. First, it describes a single case, which precludes causal inference and limits external validity. Second, outcomes were documented primarily through therapist observation, session logs, and caregiver report, and no standardized or objective outcome measures (e.g., validated scales of motor function, posture, or participation) were applied, increasing the risk of subjectivity and potential overestimation of effects. Third, baseline functional classification was only partially available from routine clinical documentation, and no formal international classification (e.g., ICF coding) was performed. Fourth, there was no blinding, no independent assessment, and no comparison condition, and possible confounders (e.g., natural maturation, concurrent interventions, changes in routines) cannot be fully excluded. Finally, follow-up beyond the immediate intervention period was limited. Accordingly, these findings should be interpreted as qualitative, hypothesis-generating observations and are not generalizable.

Future work should evaluate similar low-resource, engagement-oriented approaches using standardized outcome measures and longer follow-up.

## Conclusion

In summary, this report presents a low-cost, therapist-designed suspension-based approach associated with observable changes in engagement and task performance in one child with neurodevelopmental impairment. These observations are qualitative and hypothesis-generating and should not be generalized; however, they may inform the development of feasible, motivating interventions for pediatric rehabilitation in low-resource settings.

While the outcomes in this single case cannot be generalized, the report supports further evaluation of similar low-resource, engagement-oriented approaches using standardized outcome measures, independent assessment where feasible, and longer follow-up in small prospective cohorts.

## Data Availability

The original contributions presented in the study are included in the article/Supplementary Material, further inquiries can be directed to the corresponding author.
